# Regulation of cardiac fibrosis in mice with TAC/DOCA‐induced HFpEF by *resistin‐like molecule gamma* and *adenylate cyclase 1*

**DOI:** 10.1002/2211-5463.13813

**Published:** 2024-05-06

**Authors:** Dawei Liu, Fanling Zeng, Zhiyu Chen, Zheng Qin, Zhiqiang Liu

**Affiliations:** ^1^ The First Affiliated Hospital of Chongqing Medical University China; ^2^ Department of Cardiology, Bishan Hospital of Chongqing Bishan Hospital of Chongqing Medical University China; ^3^ Health Management Center The First Affiliated Hospital of Chongqing Medical University China; ^4^ Orthopedic Laboratory of Chongqing Medical University China; ^5^ Department of Vascular Surgery The First Affiliated Hospital of Chongqing Medical University China

**Keywords:** Adcy1, cardiac fibrosis, heart failure with preserved ejection fraction, Relmg

## Abstract

Heart failure with preserved ejection fraction (HFpEF) is one of the major subtypes of heart failure (HF) and no effective treatments for this common disease exist to date. Cardiac fibrosis is central to the pathology of HF and a potential avenue for the treatment of HFpEF. To explore key fibrosis‐related genes and pathways in the pathophysiological process of HFpEF, a mouse model of HFpEF was constructed. The relevant gene expression profiles were downloaded from the Gene Expression Omnibus database, and single‐sample Gene Set Enrichment Analysis (ssGSEA) was performed targeting fibrosis‐related pathways to explore differentially expressed genes (DEGs) in healthy control and HFpEF heart tissues with cross‐tabulation analysis of fibrosis‐related genes. Gene Ontology (GO) enrichment and Kyoto Encyclopedia of Genes and Genomes (KEGG) pathway enrichment analyses were performed on the identified fibrosis‐related genes. The two most significant DEGs were selected, and further validation was conducted in HFpEF mice. The results indicated that myocardial fibrosis was significantly upregulated in HFpEF mice compared to healthy controls, while the ssGSEA results revealed significant differences in the enrichment of nine fibrosis‐related pathways in HFpEF myocardial tissue, with 112 out of 798 DEGs being related to fibrosis. The *in vivo* results demonstrated that expression levels of *resistin‐like molecule gamma* (*Relmg*) and *adenylate cyclase 1* (*Adcy1*) in the heart tissues of HFpEF mice were significantly higher and lower, respectively, compared to healthy controls. Taken together, these results suggest that *Relmg* and *Acdy1* as well as the fibrosis process may be potential targets for HFpEF treatment.

## Graphical abstract

A mouse model of heart failure with preserved ejection fraction (HFpEF) was established to explore key fibrosis‐related genes and pathways in the HFpEF pathophysiological process. Potential candidate genes were identified by bioinformatic analyses and further validated *in vivo*. Our results suggest that *Relmg* and *Acdy1* as well as the fibrosis process may be potential targets for HFpEF treatment.

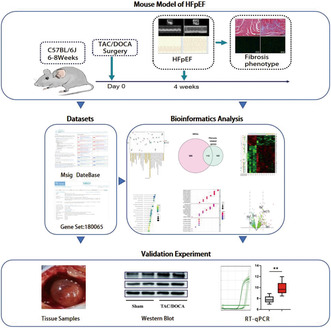

**Abbreviations**
Adcy1: adenylate cyclase 1
ANP: atrial natriuretic peptide
ATP: adenosine triphosphate
BNP: brain natriuretic peptide
cAMP: cyclic adenosine 3′,5′‐monophosphate
COL1A1: type I collagen alpha 1
COL3A1: type III collagen alpha 1
DEGs: differentially expressed genes
DOCA: deoxycorticosterone acetate
ECM: extracellular matrix
EDV: end‐diastolic volume
ESV: end‐systolic volume
FOXO: forkhead box O
GEO: Gene Expression Omnibus
GO: Gene Ontology
HE: hematoxylin–eosin
HF: heart failure
HFpEF: heart failure with preserved ejection fraction
HW: heart weight
IVSd: interventricular septum thickness in diastole
KEGG: Kyoto Encyclopedia of Genes and Genomes
LV mass: left ventricular mass
LVEF: left ventricular ejection fraction
LVFS: left ventricular fractional shortening
LVIDd: left ventricular internal dimension in diastole
LVIDs: left ventricular internal dimension in systole
LW: lung weight
NESs: normalized enrichment scores
PA: pulmonary artery
PPAR: peroxisome proliferator‐activated receptor
PWTd: posterior wall thickness in diastole
Relmg: resistin‐like molecule gamma
RT‐qPCR: reverse transcription‐quantitative polymerase chain reaction
RVSP: right ventricular systolic pressure
SPF: specific pathogen‐free
ssGSEA: single‐sample Gene Set Enrichment Analysis
SV: stroke volume
TAC: transverse aortic constriction
TAPSE: tricuspid annular plane systolic excursion
Tei index: Tei's myocardial performance index
TL: tibia length
WGA: wheat germ agglutinin

Heart failure with preserved ejection fraction (HFpEF) is a common disease in the elderly population and the incidence rate is increasing, with higher rates observed in females compared to males, characterized by inflammation, cardiac fibrosis, and microvascular dysfunction [1, 2]. Left ventricular diastolic dysfunction is a major feature of HFpEF that is driven mainly by the accumulation of reactive fibrosis in the myocardium, leading to reduced ventricular compliance and increased stiffness [3, 4, 5]. The pathophysiology of HFpEF is complex, and, unlike in patients experiencing heart failure with reduced ejection fraction, the treatments targeting neurohormonal peptide signals (e.g., angiotensin I‐converting enzyme inhibitors, angiotensin II receptor blockers, beta receptor blockers, aldosterone antagonists, and digitalis) barely improve the symptoms and prognosis of patients with HFpEF [6, 7]. Evidence suggested that reducing the pro‐fibrotic signaling can improve the outcome of HFpEF, which means that targeting reactive fibrosis is an important avenue for the treatment of HFpEF [8].

With the rapid development of bioinformatics analysis and high‐throughput sequencing technologies (including second‐generation gene sequencing), studies related to the pathophysiology of HFpEF have intensified, leading to the discovery and validation of new genes and pathways associated with this disease [9, 10]. In contrast to traditional methods, second‐generation gene sequencing allows for the quantification of large amounts of biomolecules, providing a novel approach for discovering new potential biomarkers, diagnosing and prognosticating diseases, and exploring new drug targets [11]. This method involves employing machine learning or artificial intelligence‐based algorithms to perform inductive, unbiased analysis and outputting data for the identification of key pathophysiological pathways involved in the progression of HFpEF in order to identify new therapeutic targets.

This study aimed to investigate the myocardial tissue changes in HFpEF by establishing a mouse model through minimally invasive transverse aortic constriction (TAC) surgery and deoxycorticosterone acetate (DOCA) pellet implantation. Subsequently, a notable increase in myocardial tissue fibrosis was observed in the HFpEF mice [12]. To further validate the findings, we analyzed the mRNA expression profiles of heart tissues from HFpEF mice obtained from the Gene Expression Omnibus (GEO) database targeting fibrosis‐related pathways (GSE180065) for further analysis to identify potential differentially expressed genes (DEGs) involved in the development of HFpEF. Their expression was subsequently analyzed to provide new insights for exploring biomarkers or therapeutic targets for HFpEF.

## Methods

### Experimental animals and treatments

Specific pathogen‐free (SPF) male C57BL/6J mice aged 6–8 weeks were purchased from the Experimental Animal Center of Chongqing Medical University. The mice were raised in an SPF animal experiment room with a constant temperature of 20 ± 3 °C, a humidity of 55 ± 10%, and a 12‐h light/dark cycle. The mice were randomly divided into two groups: the Sham group and the HFpEF group. The mouse model of HFpEF was constructed using the TAC/DOCA method, as described previously [12]. The mice were anesthetized and underwent either sham or TAC surgery. The TAC procedure involved inserting a 27‐gauge needle above the aortic arch, ligating it with a 5‐0 nylon suture, and then removing the needle. Finally, the DOCA pellet was placed on the chest wall. The sham surgery group underwent identical surgical procedures as the TAC/DOCA group, but without aortic arch ligation and DOCA pellet insertion. Six mice in each group were treated for 4 weeks. The method of euthanizing mice involved intraperitoneal injection of barbiturate solution, ensuring rapid and humane euthanasia. Prior to injection, mice were lightly anesthetized to minimize distress. Death was confirmed by the absence of heartbeat and respiration. All animal experiments were performed in accordance with institutional, local, and national guidelines on animal research and ethics, and were approved by the Ethics Committee of the First Affiliated Hospital of Chongqing Medical University (no. 2020‐146, no. 2020‐606).

### Echocardiography

Echocardiography was performed on the mice under anesthesia using the VisualSonics Vevo3000 imaging system (FUJIFILM VisualSonics, Toronto, Canada), and an M‐mode echocardiogram was recorded at the papillary muscle level to examine the cardiac function and ventricular structure. The left ventricular ejection fraction (LVEF), the left ventricular fractional shortening (LVFS) and the tricuspid annular plane systolic excursion (TAPSE) were measured and calculated based on the M‐mode recordings. Additionally, pulsed Doppler ultrasound mode and tissue Doppler ultrasound mode were used to determine the peak E velocity, peak e′ velocity, and pulmonary artery peak flow velocity (PA peak flow velocity). Other indices, including end‐diastolic volume (EDV), end‐systolic volume (ESV), stroke volume (SV), left ventricular mass (LV mass), and Tei's myocardial performance index (Tei index), are calculated using relevant formulas. The experiment was conducted with strict adherence to blinded protocols, with the same researcher.

### Hemodynamics

Right ventricular systolic pressure (RVSP) was measured via intercostal muscle puncture using a physiological recording system (MP150; Biopac Systems, Inc., Goleta, CA, USA). After anesthetizing the mice, the left chest ribs were exposed, and a puncture was made at the subcostal margin of the fourth intercostal space, reaching a depth of approximately 10 mm. Recordings were performed under steady‐state conditions until three stable and consecutive measurements were obtained.

### Enzyme‐linked immunosorbent assay analysis

After 4 weeks of modeling, the orbital blood samples of the mice were collected. The levels of circulating serum atrial natriuretic peptide (ANP) and brain natriuretic peptide (BNP) in the mice were detected using the corresponding enzyme‐linked immunosorbent assay kits (Quanzhou Kyobang Biotechnology Co., Ltd., Quanzhou, Fujian, China), according to the manufacturer's instructions.

### Histological examination

Samples were fixed in 4% paraformaldehyde at 4 °C overnight and dehydrated through successive washes with ethanol. The heart tissues were then sliced and subjected to hematoxylin–eosin (HE) staining to observe the general morphology of the heart tissue. Masson's trichrome staining was performed to differentiate the various tissue components. In addition, wheat germ agglutinin (WGA) staining was performed to quantify the dimensions of cardiomyocytes, and they were subsequently visualized under a microscope. Finally, we utilized image‐pro plus 6.0 software (Media Cybernetics, Rockville, MD, USA) to calculate the fibrotic area and cell cross‐sectional area in four different fields of view within each section and then performed statistical analysis based on the averaged values.

### Data resource and single‐sample gene set enrichment analysis targeting fibrosis‐related pathways

The expression profile data (GSE180065) were downloaded from the GEO database (https://www.ncbi.nlm.nih.gov/geo/) using high‐throughput sequencing. The GSE180065 dataset comprises 10 HFpEF samples and 5 control samples. The mouse C5 gene datasets were then downloaded from WEHI Bioinformatics‐mouse and human versions of the MSigDB in R format. The data were filtered based on “fibrosis,” and 30 gene sets related to fibrosis‐associated pathways were saved. To observe the enrichment of fibrosis‐related pathways, the GSE180065 data were analyzed via single‐sample gene set enrichment analysis (ssGSEA) based on 30 Gene Ontology (GO) fibrosis‐related pathways, and the score for each of the 30 pathways was calculated for each sample. The samples were divided into two groups based on “control” and “HFpEF,” and statistical analysis was performed across the two groups. The normalized enrichment scores (NESs) of the fibrosis subsets with significant differences (*P* < 0.05) were used for the following analysis.

### Identification of DEGs and intersection analysis

The DEGs across the HFpEF group and the control group in the GSE180065 dataset were analyzed using the deseq2 package in r (version, 1.38.2). The threshold standard of adjusted *P*‐value ≤ 0.05 was set to select DEGs with statistical differences. To visualize the results, the heatmap package in r was used for hierarchical clustering, and the regions in which the DEGs were mainly concentrated were highlighted. The genes in the pathways with statistical differences from the previous ssGSEA were extracted and were found to intersect with the previously identified DEGs to ultimately obtain DEGs related to fibrosis.

### Function and pathway enrichment analysis of DEGs

To further observe the functions and pathways of DEGs related to fibrosis, GO and Kyoto Encyclopedia of Genes and Genomes (KEGG) analyses were performed. Annotations of the cellular components, biological processes, and molecular functions of the DEGs were identified via GO enrichment analysis, while the pathways and the associated functions of the gene clusters were determined using KEGG pathway analysis. The r clusterprofiler package (version, 4.6.0) was used to explore the GO enrichment and KEGG pathway analysis.

### Reverse transcription‐quantitative polymerase chain reaction

Two key genes were selected for validation using reverse transcription‐quantitative polymerase chain reaction (RT‐qPCR), and the total RNA was extracted from the heart tissue using the TRIzol™ reagent (Takara Bio Inc., Kusatsu, Shiga, Japan), according to the manufacturer's protocol. Complementary DNA was generated using a commercial kit (Takara). The gene expression levels were then quantified via real‐time PCR utilizing the CFX Connect Real‐Time PCR System (Bio‐Rad Laboratories, Inc., Hercules, CA, USA). The expression levels were normalized to glyceraldehyde 3‐phosphate dehydrogenase, which served as an internal control, using the $2^{-\Delta \Delta C_{t}}$ method. The *resistin‐like molecule gamma* (*Relmg*) primers were as follows: forward 5′‐CTTGCCAATCGAGATGACTGT‐3′, and reverse 5′‐AGTCTGCCTGAAGCCGTGATA‐3′. The *adenylate cyclase 1* (*Adcy1*) primers were as follows: forward 5′‐AAACACAGTCAATGTGGCCAGTCG‐3′, and reverse 5′‐ACTTTGCCTCTGCACACAAACTGG‐3′. The *Collagen I* primers were as follows: forward 5′‐ATGCCTGGTGAACGTGGT‐3′, and reverse 5′‐AGGAGAGCCATCAGCACCT‐3′.

### Western blot analysis

The total protein was extracted using radioimmunoprecipitation assay buffer containing phenylmethylsulfonyl fluoride (Beyotime Biotechnology, Shanghai, China). A bicinchoninic acid protein assay kit (Beyotime Biotechnology) was used to determine the protein concentrations. Loading dye (Beyotime Biotechnology) was added to the samples prior to heating at 100 °C for 10 min. An equal amount of protein was separated via sodium dodecyl‐sulfate polyacrylamide gel electrophoresis and transferred onto polyvinylidene difluoride membranes. The membranes were blocked in 5% skimmed milk for 2 h, followed by an overnight incubation at 4 °C with the following primary antibodies: Adcy1 (1 : 500; ABclonal Technology, Wuhan, Hubei, China), Relmb (1 : 1000; ABclonal), Collagen I (1 : 1000; ABclonal), Vimentin (1 : 1000; ABclonal), and α‐sma (1 : 1000; ABclonal), respectively. Then, the membranes were incubated with horseradish peroxidase‐conjugated secondary antibodies (1 : 5000; ABclonal) before being subjected to protein signal detection using the enhanced chemiluminescence reagent.

### Statistical analysis

Data analysis was performed using spss 22.0 software (IBM Corporation, Armonk, NY, USA). The data were expressed as the mean ± standard deviation. The Student's *t‐*test was employed to compare the differences between two groups, while one‐way analysis of variance was performed followed by the Brown–Forsythe and Welch tests to distinguish the differences among multiple comparisons. The differences were considered statistically significant at a level of *P* < 0.05.

## Results

### Construction of the mouse model of HFpEF

**Fig. 1:**
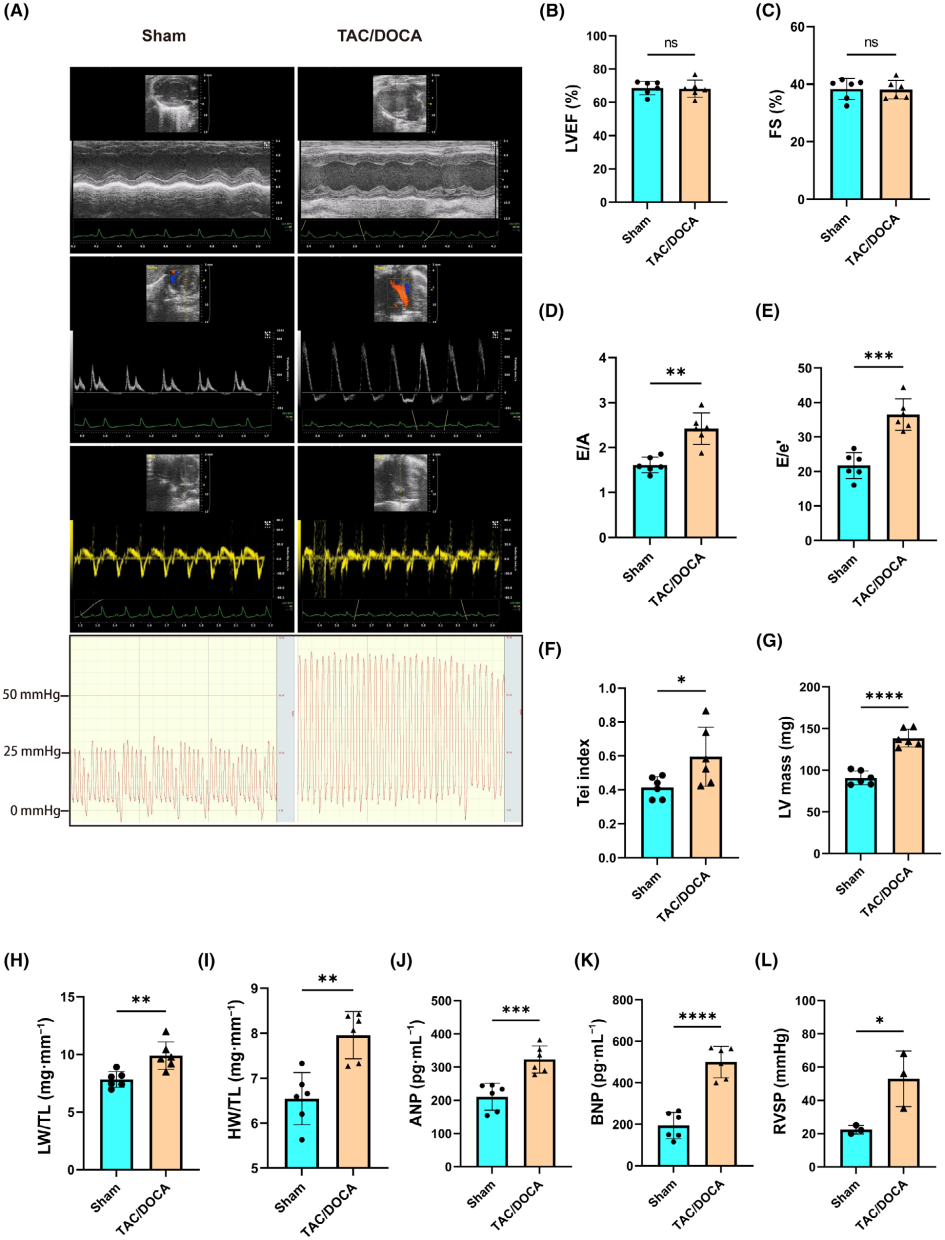
The key changes observed in both tissue morphometry, echocardiographic parameters and hemodynamics in the TAC/DOCA group that are indicative of HFpEF occurred at week 4. (A) Representative left ventricular M‐mode echocardiographic, pulsed‐wave Doppler, tissue Doppler tracings and RVSP measurement. (B) Percentage of LVEF; *n* = 6 mice per group. (C) Percentage of FS; *n* = 6 mice per group. (D) Ratio between mitral E wave and A wave (E/A); *n* = 6 mice per group. (E) Ratio between mitral E wave and e′ wave (E/e′); *n* = 6 mice per group. (F) Tei's myocardial performance index (Tei index); *n* = 6 mice per group. (G) The left ventricular mass estimated by echocardiography (LV mass); *n* = 6 mice per group. (H, I) Ratio of lung weight to tibia length (LW/TL) and ratio of heart weight to tibia length (HW/TL); *n* = 6 mice per group. (J, K) Serum ANP and BNP levels were detected via enzyme‐linked immunosorbent assay; *n* = 6 mice per group. (L) Statistical results of RVSP; *n* = 3 mice per group. Two‐tailed unpaired Student's *t*‐test; *P < 0.05, **P < 0.01, ***P < 0.001, ****P < 0.0001. ANP, atrial natriuretic peptide; BNP, brain natriuretic peptide; DOCA, deoxycorticosterone acetate; FS, fractional shortening; HW, heart weight; LVEF, left ventricular ejection fraction; LW, lung weight; ns, not significant; RVSP, right ventricular systolic pressure; TAC, transverse aortic constriction; TL, tibia length.

**Fig. 2:**
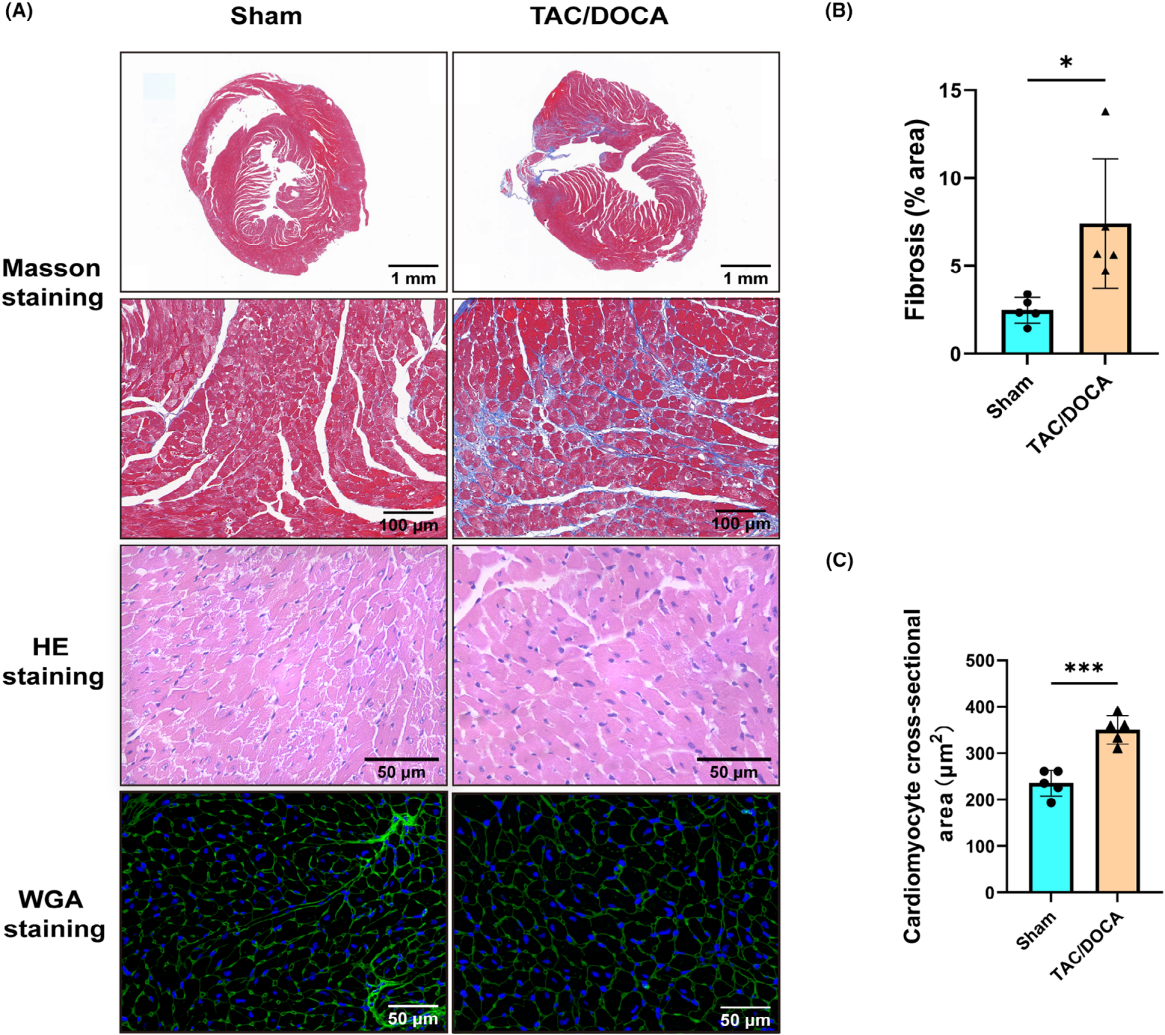
TAC/DOCA mice showed cardiac fibrosis and cardiomyocyte hypertrophy after 4 weeks of modeling. (A) Histological images of cardiac sections stained with Masson, HE, and WGA staining. Scale bar: 1 mm (first row), 100 μm (second row), 50 μm (third row), 50 μm (fourth row); *n* = 5 mice per group. (B) Percentage of fibrosis area in Masson's stained transversal sections; *n* = 5 mice per group. (C) Cardiomyocyte cross‐sectional area in WGA stained transversal sections; *n* = 5 mice per group. Two‐tailed unpaired Student's *t*‐test; *P < 0.05, ***P < 0.001.

To explore the changes in the cardiac structure after HFpEF, a HFpEF mouse model was constructed using the TAC/DOCA method, and the cardiac systolic and diastolic functions of HFpEF were evaluated via morphometry and echocardiography (Table 1). Compared with the Sham group, the TAC/DOCA group exhibited no significant changes in the systolic function parameters of LVEF and LVFS. However, parameters reflecting diastolic function such as E/A, E/e′, and Tei index showed significant deterioration (Fig. 1A–F). Additionally, the TAC/DOCA group displayed elevated cardiac hypertrophy index, pulmonary congestion index, right ventricular systolic pressure (RVSP), as well as increased levels of serum ANP and BNP (Fig. 1G–L). These findings suggest the successful establishment of a HFpEF model, consistent with previous studies. To further explore changes in the cardiac structure, HE staining, WGA staining, and Masson staining of cardiac slices were performed, and the results indicated that the myocardial fibrosis and cardiomyocyte cross‐sectional area of the HFpEF mice was significantly aggravated compared to those of the Sham group (Fig. 2A–C).

**Table 1:** Tissue morphometry and echocardiographic parameters at 4 weeks after TAC/DOCA surgery. Results are presented as mean ± SD. Two‐tailed unpaired Student's *t*‐test. *n* = 6 mice per group. A, peak Doppler blood infow velocity across mitral valve during late diastole; Body wt, body weight; E, peak Doppler blood infow velocity across mitral valve during early diastole; e′, peak tissue Doppler of myocardial relaxation velocity at mitral valve annulus during early diastole; FS, fractional shortening; HW, heart weight; IVSd, interventricular septum thickness in diastole; LV + S, left ventricular + septal mass; LV, left ventricle; LVEDV, LV end‐diastolic volume; LVEF, LV ejection fraction; LVESV, LV end‐systolic volume; LVIDd, left ventricular internal dimension in diastole; LVIDs, left ventricular internal dimension in systole; LW, lung weight; PA peak flow velocity, pulmonary artery peak flow velocity; PWTd, posterior wall thickness in diastole; SV, stroke volume; TAPSE, tricuspid annular plane systolic excursion; TL, tibia length.

	Sham (*n* = 6)	TAC/DOCA (*n* = 6)	*P*‐value
Tissue morphometry			
Body wt (g)	22.92 ± 0.61	23.62 ± 1.83	0.395
TL (mm)	17.42 ± 0.66	16.98 ± 0.16	0.152
HW/TL (mg·mm^−1^)	6.54 ± 0.58	7.96 ± 0.53	0.001
HW/Body wt (mg·g^−1^)	4.98 ± 0.5	5.76 ± 0.69	0.048
LW/TL (mg·mm^−1^)	7.83 ± 0.69	9.9 ± 1.2	0.004
LW/Body wt (mg·g^−1^)	5.94 ± 0.35	7.1 ± 0.38	< 0.001
(LV + S)/TL (mg·mm^−1^)	4.01 ± 0.29	5.15 ± 0.31	< 0.001
Echocardiography			
LVEF (%)	68.66 ± 4.08	68.23 ± 5.11	0.874
FS (%)	38.36 ± 3.64	38.11 ± 3.22	0.902
E/A	1.61 ± 0.18	2.42 ± 0.35	0.001
E/e′	21.73 ± 3.76	36.5 ± 4.56	< 0.001
LVIDd (mm)	3.37 ± 0.15	3.78 ± 0.2	0.003
LVIDs (mm)	2.12 ± 0.31	2.49 ± 0.14	0.023
LVEDV (μL)	46.44 ± 4.99	61.41 ± 7.83	0.003
LVESV (μL)	15.21 ± 4.97	22.12 ± 2.99	0.015
SV (μL)	31.23 ± 5.36	39.28 ± 7.1	0.051
IVSd (mm)	1.09 ± 0.09	1.32 ± 0.04	< 0.001
PWTd (mm)	0.82 ± 0.04	0.94 ± 0.04	< 0.001
LV mass (mg)	90.97 ± 8.23	138.66 ± 10.53	< 0.001
Tei index	0.41 ± 0.06	0.6 ± 0.17	0.038
TAPSE	1.06 ± 0.11	0.7 ± 0.186	0.002
PA peak flow velocity (mm·s^−1^)	504.78 ± 67.18	689.49 ± 71.55	0.001

### ssGSEA targeting fibrosis‐related pathways

**Fig. 3:**
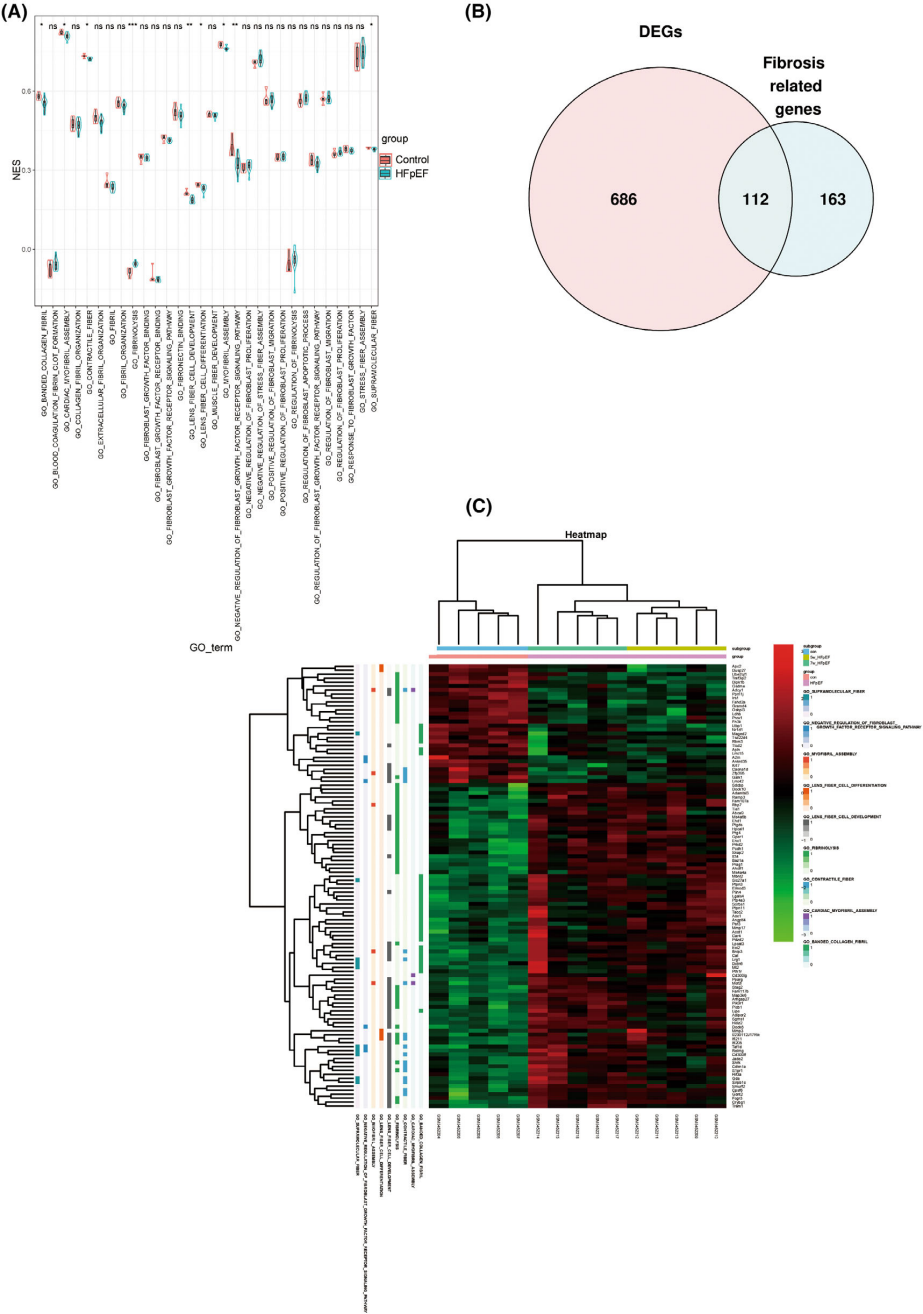
The ssGSEA targeting fibrosis and identification of DEGs. (A) The ssGSEA method targeted nine pathways associated with fibrosis. *P < 0.05, **P < 0.01, ***P < 0.001. (B) Identification of overlapping DEGs in the dataset and fibrosis‐associated genes. (C) The heat map shows all the differentiated genes classified as nine fibrosis‐related pathways with significant differences.

To further confirm the fibrosis changes in the myocardium of HFpEF mice, ssGSEA was performed to observe the expression of genes associated with fibrosis‐related pathways in the HFpEF mice. The results indicated that the pathways with significant differences between the two groups were mainly enriched in nine fibrosis‐related pathways, including the banded collagen fibril, cardiac myofibril assembly, contractile fiber, fibrinolysis, lens fiber cell development, lens fiber cell differentiation, myofibril assembly, negative regulation of the fibroblast growth factor receptor signaling, and supramolecular fiber pathways (Fig. 3A).

### Identification of DEGs

A total of 798 DEGs were identified across the control and HFpEF groups in the merged dataset. Next, the fibrosis‐associated genes in pathways with significant differences were cross‐referenced with the DEGs, and 112 fibrosis‐associated DEGs were then screened. Among these DEGs, 82 genes were upregulated and 30 were downregulated. These differential fibrosis‐related DEGs were categorized into the nine fibrosis‐related pathways with significant differences, and they were subsequently presented in a heat map. The details of the 112 DEGs are shown in Fig. 3B,C.

### Basic interpretation of 112 DEGs via GO and KEGG analyses

**Fig. 4:**
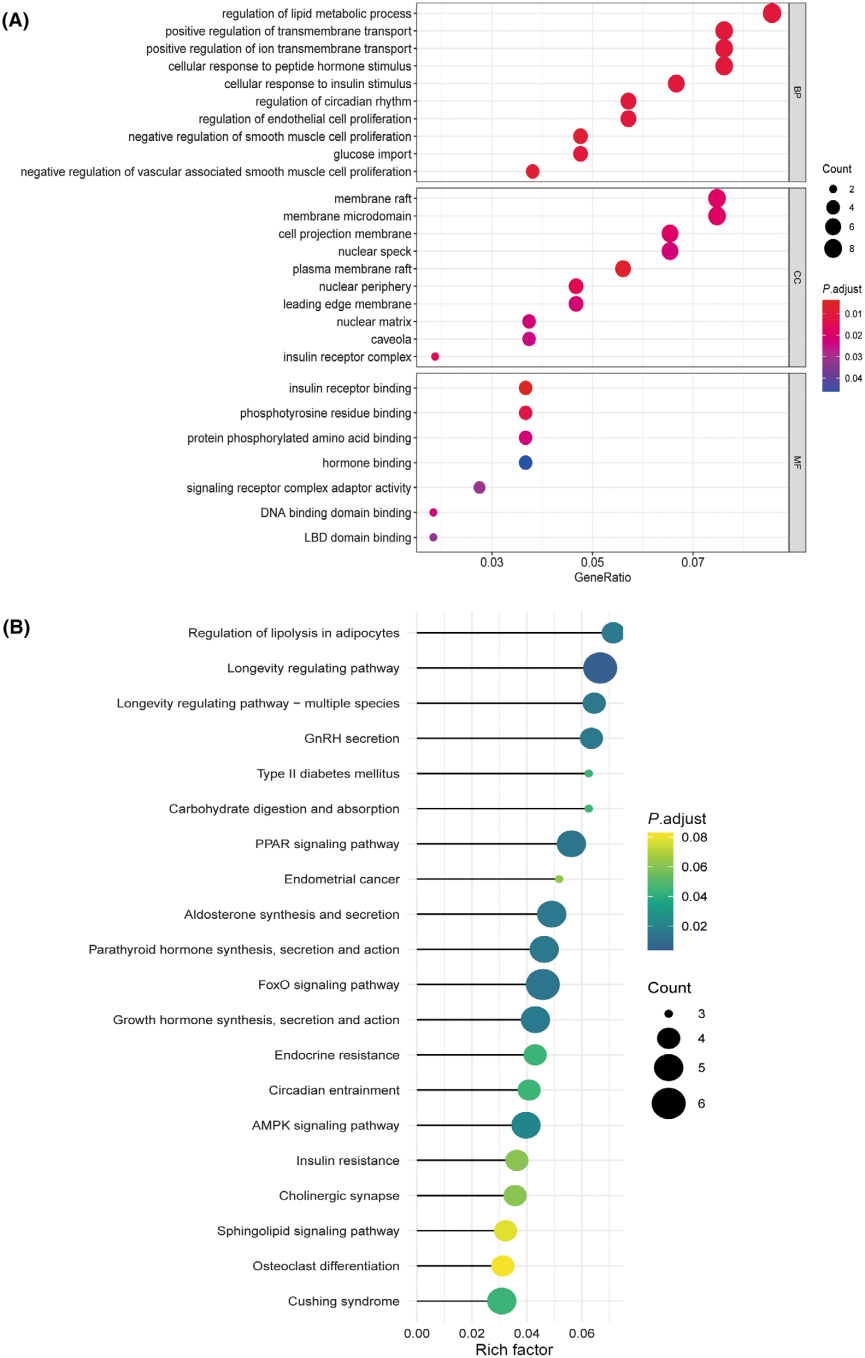
The GO enrichment and KEGG pathway analysis. (A) The GO biological processes, cellular components, and molecular functions enrichment analysis on overlapping DEGs. (B) Enriched KEGG pathways based on total overlapping DEGs.

The GO analysis annotated the DEGs as biological processes, cellular components, or molecular functions. The DEGs related to biological processes were primarily enriched in regulation of the lipid metabolic process, regulation of endothelial cell proliferation, negative regulation of smooth muscle cell proliferation, and negative regulation of vascular associated smooth muscle cell proliferation. Those related to cellular components were involved in the nuclear matrix and the insulin receptor complex. Meanwhile, the genes related to molecular function primarily participated in insulin receptor binding, phosphotyrosine residue binding, and protein phosphorylated amino acid binding. The KEGG analysis revealed that the main signaling pathways that were enriched were related to the longevity regulating pathway, peroxisome proliferator‐activated receptor (PPAR) signaling pathway, and forkhead box O (FOXO) signaling pathway, as shown in Fig. 4A,B.

### *In vivo* model validation

**Fig. 5:**
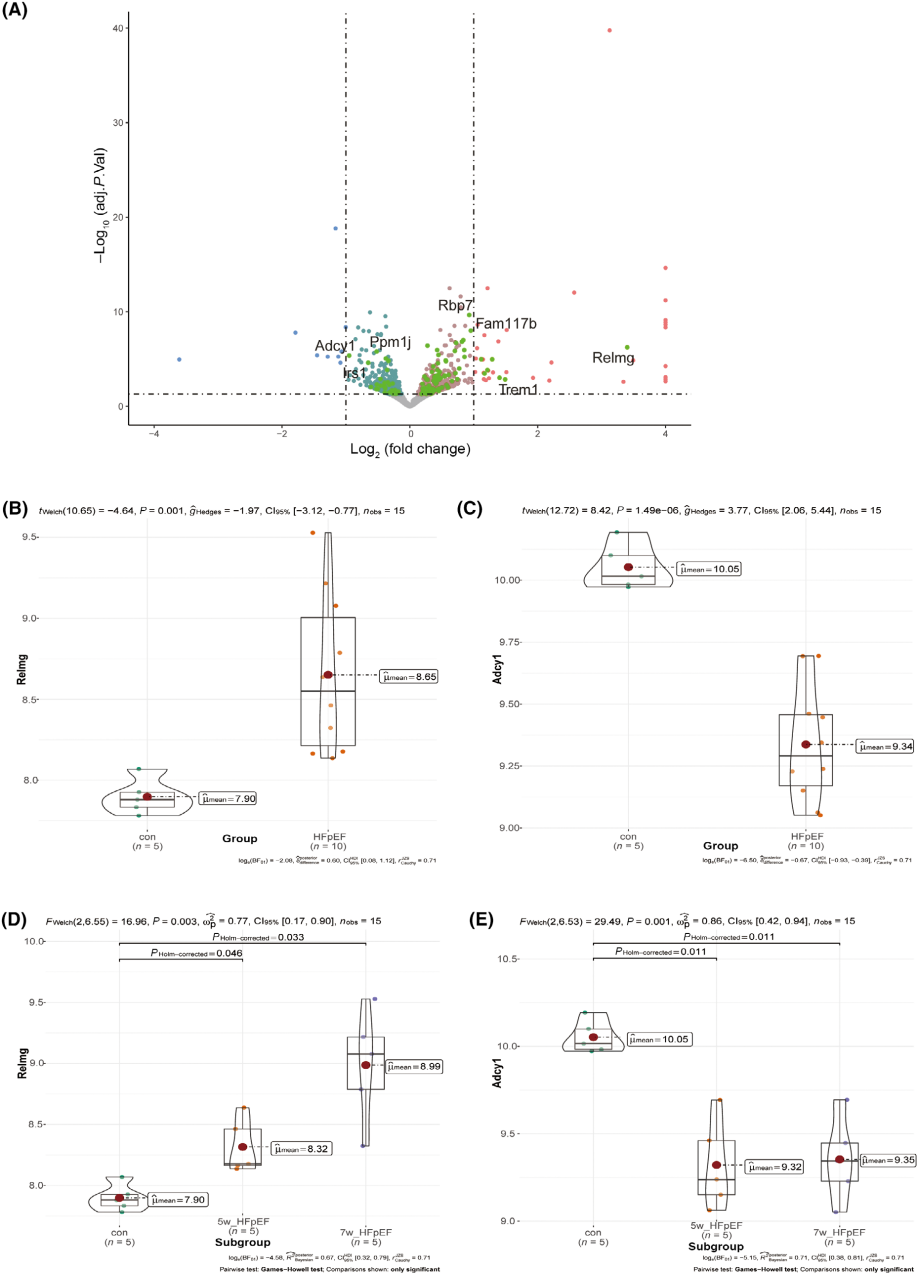
Screening of differential genes. (A) Expression profiles of overlapping genes are visualized by volcano plots. (B) The *Relmg* was upregulated in the HFpEF group compared with in the control group. (C) The *Adcy1* was significantly downregulated in the HFpEF group compared with in the control group. (D) The expression of *Relmg* in the HFpEF group was significantly higher than that in the control group at 5 and 7 weeks. (E) The expression of *Adcy1* in the HFpEF group was significantly lower than that in the control group at 5 and 7 weeks. (B, C) Two‐tailed unpaired *t*‐test with Welch's correction. (D, E) One‐way analysis of variance followed by Brown–Forsythe and Welch tests. The *P*‐values are shown above the graphs or *n* zig‐zag lines.

**Fig. 6:**
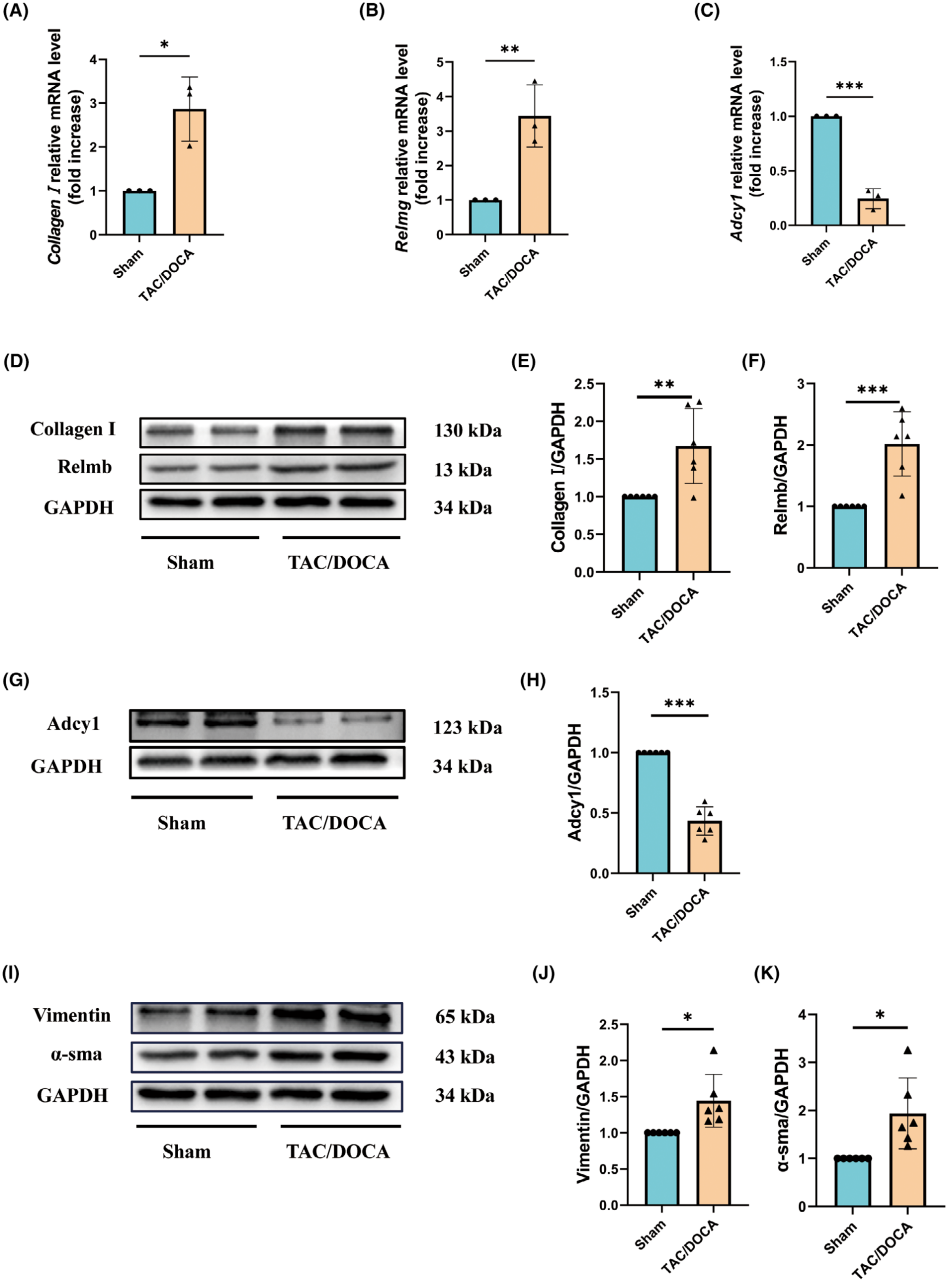
Expression levels of genes and proteins related to myocardial fibrosis in each group of mice. (A–C) Relative mRNA expression levels of *Collagen I*, *Relmg*, and *Adcy1* in mouse ventricular myocytes isolated from the sham or TAC/DOCA mice; *n* = 3 mice per group. (D–K) The protein expression of collagen I, Relmb, Adcy1, Vimentin and α‐sma determined by western blot analysis in mouse ventricular myocytes isolated from the sham or TAC/DOCA mice; *n* = 6 mice per group. Two‐tailed unpaired Student's *t*‐test. *P < 0.05, **P < 0.01, ***P < 0.001.

The genes with the highest and lowest differential expression levels were selected based on the |log_2_‐foldchange| of the fibrosis‐related DEGs: *Relmg* (logFC = 3.4, corrected *P*‐value = 5.79E‐6) and *Adcy1* (logFC = 0.94928, corrected *P*‐value = 4.32E‐6). As shown in Fig. 5A–E, the expression of *Relmg* was upregulated over time in the HFpEF group compared with that in the control group, while the expression of *Adcy1* was significantly downregulated. Using the established HFpEF mouse model, the expression of these two key genes in the myocardial tissues of the control and HFpEF mice were further verified via RT‐qPCR and western blotting, which not only validated the results of the bioinformatics analysis but also revealed the consistency of expression between the RNA and protein levels of these two genes. As shown in Fig. 6, the gene and protein levels of *Adcy1* were significantly decreased, while those of *Collagen I* were significantly increased in the HFpEF group compared with those in the control group (*P* < 0.05). Meanwhile, the gene expression level of *Relmg* and the protein expression level of Relmb, which is homologous to Relmg, were significantly increased in the HFpEF group compared with those in the control group (*P* < 0.05). The protein levels of α‐sam and Vimentin were significantly increased in the HFpEF group compared with those in the control group (*P* < 0.05). The above results were consistent with the bioinformatics analysis results.

## Discussion

Heart failure with preserved ejection fraction is a clinical syndrome associated with a poor quality of life, high utilization of healthcare resources, and premature mortality [13, 14, 15]. However, in the majority of studies, the proportion of HFpEF diagnoses is close to 50% of all diagnoses of HF, and the overall incidence of HFrEF declines, accompanied by a concurrent increase in the prevalence of HFpEF [16, 17]. Left ventricular diastolic dysfunction plays a fundamental role in the pathophysiology of HFpEF, and associated with increased left ventricular stiffness. While the underlying mechanism is not fully understood, the increased content of myocardial collagen is an important factor contributing to increased myocardial stiffness [18, 19]. Studies have shown that maintaining the homeostasis of fibroblast‐derived and myofibroblast‐derived collagen is particularly important for cardiac diastolic function [20, 21]. Fibrosis is characterized by expansion and remodeling of the extracellular matrix (ECM) as well as the upregulated expression of type I collagen alpha 1 (COL1A1), type III collagen alpha 1 (COL3A1), etc. [22, 23]. Increasing evidence suggests that an excessive ECM, especially cardiac fibrosis characterized by collagen deposition, affects the clinical course and outcome of HFpEF patients [24, 25]. The above findings suggest that fibrosis plays an important role in the pathological process of HFpEF; therefore, studying fibrosis‐related molecules will be helpful for discovering targets to treat HFpEF.

In this study, bioinformatics analysis was used to explore the biomarkers and pathological processes implicated in the HFpEF process. It was found that the fibrosis of cardiac tissue in the HFpEF mice was significantly increased. To verify this finding, DEGs were analyzed using microarray data and bioinformatics methods targeting fibrosis, and a total of 112 DEGs associated with fibrosis were identified. We then performed GO and KEGG pathway enrichment analyses on the 112 DEGs, and the results revealed that the DEGs were mainly involved in the negative regulation of endothelial cell proliferation and smooth muscle cell proliferation. In addition, the KEGG analysis results indicate that the DEGs are mainly related to the longevity regulating pathway, PPAR signaling pathway, and FOXO signaling pathway. Finally, the genes with the highest upregulated and downregulated |log_2_‐foldchange| values (*Relmg* and *Adcy1*, respectively) were selected and further validated via RT‐qPCR and western blotting, and it was found that the relative expression levels of *Relmg* and *Adcy1* matched the expression trends displayed in our bioinformatics analysis.

The RELM family, consisting of four members (*Relma*, *Relmb*, *resistin*, and *Relmg*) in rodents, is highly homologous and plays an important role in the progression of fibrotic diseases [26, 27]. Among them, *Relma* and *Relmb* are closely associated with cell proliferation, collagen synthesis, and ECM production [26]. Studies have shown that *Relma* knockout inhibits the proliferation of hypoxia‐induced pulmonary nonhematopoietic progenitor cells in mice [28], while it also has been demonstrated that the inhibition of *Relmb* attenuates transforming growth factor‐β‐induced pulmonary endothelial cell proliferation and endothelial‐mesenchymal transition [29]. Moreover, *Relma* and *Relmb* have been found to be highly expressed in bleomycin‐induced pulmonary fibrosis and to participate in the development of this condition, while the inhibition of *Relma* or *Relmb* reduces the expression of ECM remodeling genes, such as *Col1a1*, *Col3a1*, and *α‐smooth muscle actin*, thereby inhibiting the progression of pulmonary fibrosis [30, 31, 32]. However, few studies on *Relmg* exist. The findings of the present study suggest that *Relmg* may play an important role in the progression of fibrotic disease.

Meanwhile, *Adcy1*, a member of the adenylate cyclase family, catalyzes the generation of cyclic adenosine 3′,5′‐monophosphate (cAMP) from adenosine triphosphate (ATP), which interacts with protein kinase A and AMP‐activated guanine exchange factors to participate in cell differentiation, cell proliferation, apoptosis and endothelial‐mesenchymal transition [33, 34]. In addition, Bergmeier *et al*. [35] have found that *Adcy1* negatively regulates the transformation of fibroblasts to myofibroblasts and inhibits excessive ECM deposition during fibrotic repair following skin injury. It also has been demonstrated that *Adcy1* is significantly downregulated in the lungs of mice with idiopathic pulmonary fibrosis and that the administration of asiaticoside upregulated *Adcy1* expression and activates the cAMP/Rap1 signaling pathway, thereby attenuating pulmonary fibrosis [36]. Moreover, studies have shown that in rats with heart failure, the exacerbation of myocardial fibrosis was accompanied by the upregulation of the fibrosis‐promoting gene *Fgfr2* and the downregulation of *Adcy1*; while following intervention with Qishen granules, myocardial fibrosis was attenuated with the downregulation of *Fgfr2* and the upregulation of *Adcy1* [37]. In the present study, it was also found that *Adcy1* was significantly downregulated in the myocardium of the HFpEF mice, accompanied by increased myocardial fibrosis. The above results suggest that *Adcy1* plays an important role in the process of fibrosis.

Thus far, there has been limited research targeting fibrosis‐related genes when analyzing HFpEF. Nevertheless, this study has several limitations. First, since the Relmg antibody cannot be obtained in China and could not be imported from abroad due to the COVID‐19 pandemic, the level of Relmb, which is homologous to Relmg, was measured instead. Second, due to the ethical issues pertaining to human research, it was difficult for us to collect human heart tissues from HFpEF patients and perform subsequent validation. Furthermore, the expression of only two genes in animals was validated, and further relevant molecular biology experiments will be conducted in future research to expand the exploration of the regulatory role of these genes in fibrosis‐related pathways.

## Conclusion

Myocardial fibrosis plays an important role in the pathophysiological process of HFpEF by promoting unfavorable ventricular remodeling and increasing myocardial stiffness, thereby inducing diastolic dysfunction. This study found a large number of fibrosis‐related DEGs in the myocardial tissues of HFpEF mice compared with those of the healthy controls, among which *Relmg* and *Adcy1* play important roles in the pathogenesis of HFpEF. The bioinformatics analyses and animal experiments all revealed that *Relmg* promotes this disease, while *Adcy1* has an inhibitory effect. These results suggest that targeting fibrosis may play a positive role in reversing the progression of HFpEF.

## Conflict of interest

The authors declare no conflict of interest.

### Peer review

The peer review history for this article is available at https://www.webofscience.com/api/gateway/wos/peer‐review/10.1002/2211‐5463.13813.

## Author contributions

All authors made a significant contribution to the work reported, be it in terms of conception, study design, execution, acquisition of data, bioinformatics analysis, and interpretation, or in all these areas in terms of assisting with the drafting, revising or critically reviewing the article, final approval of the version to be published, agreeing on the journal to which the article will be submitted, and agreeing to be accountable for all aspects of the work. The authors take full responsibility for all aspects of the reliability and freedom from bias of the data presented and their discussed interpretation.

## Data Availability

The datasets generated and analyzed during the current study are available from the corresponding author upon reasonable request.
